# Validation of the comprehensive assessment of acceptance and commitment therapy processes for youth: The CompACT‐Y

**DOI:** 10.1002/jcv2.12271

**Published:** 2024-08-11

**Authors:** Alex Morey, Victoria Samuel, Matthew Lewis, Marc Williams

**Affiliations:** ^1^ South Wales Doctoral Programme in Clinical Psychology Cardiff University Cardiff UK; ^2^ Specialist Perinatal Services Swansea Bay UHB Tonna Hospital Tonna UK

**Keywords:** acceptance and commitment therapy, adolescence, process measure, psychological flexibility, ‘psychometric validation’

## Abstract

**Background:**

Acceptance and Commitment Therapy (ACT) is a transdiagnostic approach which aims to increase psychological flexibility. Higher psychological flexibility has been associated with reduced psychological distress, mental health symptoms and improvements in well‐being and functioning. Reviews of ACT for children and young people (CYP) indicate it shows potential as an effective treatment for a range of difficulties, however a comprehensive measure of psychological flexibility processes does not exist for CYP. Following revision of the adult Comprehensive assessment of Acceptance and Commitment Therapy Processes (CompACT), through cognitive interviewing with adolescents and consultation with ACT experts, the present study aimed to assess the factor structure and validity of the revised youth measure (the CompACT‐Y).

**Method:**

The CompACT‐Y measure was administered alongside measures of ACT processes, mental health and well‐being to 334 young people across six UK schools, to assess for convergent and concurrent validity.

**Results:**

Exploratory factor analysis indicated a 19‐item three‐factor structure was the most stable, with all items loading above 0.50. The CompACT‐Y correlated with measures of psychological flexibility (*r* = ‐0.64 – 0.66), mental health (*r* = −0.58 – −0.66), well‐being (*r* = 0.57–0.65) and behaviour (*r* = −0.63) as expected, indicating acceptable convergent and concurrent validity.

**Conclusions:**

The CompACT‐Y appears to be a valid and reliable measure of psychological flexibility in young people. Further research is needed to replicate the findings and confirm factor structure, validity and reliability, particularly in younger adolescents and those from diverse backgrounds. The CompACT‐Y offers a promising tool to improve the methodological rigour of ACT studies in young people, and has implications for the use of ACT in clinical practice.

**Key points**

- The study aimed to assess the factor structure and validity of the Comprehensive Measure of Acceptance and Commitment Therapy proccesses for Youth (CompACT‐Y), a youth version of an adult psychological flexibility measure.
- Exploratory factor analysis identified a stable 19‐item three‐factor structure for CompACT‐Y, consistent with the adult version and psychological flexibility theory.
- The CompACT‐Y demonstrated good convergent and concurrent validity, indicated by significant correlations with other measures of psychological flexibility, mental health and well‐being.
- The Compact‐Y is a valid and reliable measure for assessing psychological flexibility in young people. Further research is recommended to replicate findings and confirm the measure's factor structure, validity, and reliability across more diverse and younger adolescent populations.
- The CompACT‐Y holds potential to enhance methodological rigour in ACT studies and clinical practice involving young people.

## INTRODUCTION

Psychological flexibility is understood to be a dynamic trait which can be used in different contexts to change or persist with behaviour in the direction of an individual's values (Doorley et al., 2020). Psychological flexibility encompasses six core processes: acceptance; cognitive defusion; contacting the present moment; values; self‐as‐context and committed action (Hayes et al., 2006). These processes have been combined into three core components, the ‘Triflex’ (Harris, 2019); being present (contact present moment, self‐as‐context), doing what matters (values, committed action) and opening up (acceptance, defusion).

Acceptance involves recognising and allowing negative thoughts, feelings, and experiences, and cognitive defusion refers to the act of observing one's thoughts and feelings without becoming entangled by their content and meaning. Contacting the present moment involves being mindful of one's current internal experiences, and self‐as‐context is a way of viewing internal experiences from an observer perspective. Values refer to identifying and following important principles in one's life, whilst committed action is the process of actively working towards these values despite difficult or adverse experiences (Hayes et al., 2011). When an individual is psychologically flexible, they embrace challenging thoughts and emotions, engage with the present moment, using mindful awareness and a detached perspective on internal experiences, and align their actions with chosen values for meaningful living (Harris, 2006).

Increasing psychological flexibility has significant implications for mental health and well‐being. Research has suggested psychological flexibility can moderate the negative effects of life stressors on mental and physical health, as well as overall well‐being (Fonseca et al., 2020; Gloster et al., 2017; Landi et al., 2020). Tyndall et al. (2020) found that those scoring highest on psychological flexibility reported the lowest levels of psychological distress, and other research has associated higher psychological flexibility with greater well‐being, lower distress, and effective coping strategies (Dawson & Golijani‐Moghaddam, 2020).

### Acceptance and commitment therapy for children and young people (CYP)

Acceptance and Commitment Therapy (ACT) is a transdiagnostic therapeutic approach that conceptualises distress as resulting from counterproductive attempts to avoid or suppress unwanted internal experiences such as our thoughts, feelings, and sensory sensations (known as experiential avoidance), alongside decreased involvement in meaningful activities (Hayes et al., 1999). The main aim of ACT interventions is to minimise the impact of psychological distress on engaging in valued action by promoting psychological flexibility.

Two reviews of ACT for CYP were conducted in 2020. Harris and Samuel (2020) found that ACT interventions to prevent or treat mental health difficulties in CYP reduced symptoms across a range of difficulties including anxiety, depression, eating disorders and OCD. Fang and Ding (2020) conducted a meta‐analysis on randomised controlled trials (RCTs) of ACT for CYP. The review concluded that ACT significantly improved mental health outcomes compared to control conditions. ACT effectiveness was comparable to CBT and active controls, and ACT surpassed control conditions in enhancing quality of life and psychological well‐being. However, both reviews highlighted methodological limitations of reviewed studies including small sample sizes and brief follow‐up periods. Heterogeneous methodologies and disparate measures of psychological flexibility subprocesses made comparison and data integration across CYP studies challenging.

### Measuring psychological flexibility in CYP

As research into ACT and psychological flexibility expands, it is necessary to have a robust measure to determine whether ACT interventions result in changes of psychological flexibility as theoretically expected. Cherry et al. (2021) highlighted challenges in defining and measuring psychological flexibility and inflexibility due to its multifaceted conceptualisation, varied terminology and different definitions.

To evaluate the processes of change in how ACT interventions enhance psychological flexibility, a broad measure of psychological flexibility is essential. Some research has shown that greater psychological flexibility in later therapy sessions predicts fewer symptoms at follow‐up (Fledderus et al., 2013). However, identifying which aspects of psychological flexibility contribute to these significant changes requires measuring each subprocess in every treatment session. A comprehensive measure for CYP would enable analysis of psychological flexibility components as predictors of therapeutic outcomes. This approach would support both research and clinical practice by determining how well ACT interventions target the core processes of psychological flexibility, and by enabling the tailoring of interventions based on session‐by‐session measurements.

Multiple measures currently exist to assess psychological flexibility in CYP, focusing on specific subprocesses like experiential avoidance, cognitive fusion, or mindfulness. Examples include the Avoidance and Fusion Questionnaire‐Youth (AFQ‐Y; Greco et al., 2008) and the Child and Adolescent Mindfulness Measure (CAMM; Greco et al., 2011). The AFQ‐Y and its shorter version, AFQ‐Y8, measure psychological inflexibility by assessing experiential avoidance and cognitive fusion. However, the AFQ‐Y was derived from items on the Acceptance and Action Questionnaire (AAQ‐II; Bond et al., 2011) for adults, which has faced criticism for its association with distress rather than acceptance, and poor construct validity for experiential avoidance (Rochefort et al., 2018; Wolgast, 2014). A review of the AFQ‐Y8 (Lewis, 2020) also highlighted its limited content validity due to a lack of high‐quality studies confirming item accuracy and low test‐retest reliability.

Research studies can require up to eight different outcome measures to evaluate the effectiveness of ACT interventions for CYP: target symptom or well‐being measures and multiple psychological flexibility process measures (Petts et al., 2017; Swain et al., 2015; Timko et al., 2015). This can be burdensome, repetitive, and confusing for participants (Demkowicz et al., 2020). Furthermore, the lack of a comprehensive measure of psychological flexibility for CYP has clinical implications. The use of outcome measures has increased in CYP services since the implementation of the CYP Improving Access to Psychological Therapies (CYP‐IAPT) framework (Wolpert et al., 2015). However, the need for multiple measures to evaluate changes in psychological flexibility presents a barrier to completion and places additional demands on clinicians and CYP. A single measure of psychological flexibility specifically designed for CYP would alleviate clinician burden and facilitate the assessment of ACT processes in clinical practice.

Newer psychological flexibility measures for CYP include the Psy‐Flex‐A (Soares et al., 2023), a European Portuguese adolescent version of the six‐item Psy‐Flex (Gloster et al., 2021). Adapted for comprehensibility based on feedback from 10 adolescents, PsyFlex‐A underwent validation for factor structure and convergent validity in a larger sample. However, it lacks validation in English‐speaking populations and measures each subprocess with only one item, providing an overall score without assessing specific subprocesses. Another CYP measure, the Children's Psychological Flexibility Questionnaire (CPFQ; Bachmann et al., 2021), validated in CYP with autism, showed correlations with experiential avoidance, mindfulness, and psychological inflexibility measures. CPFQ's validation has been extended to neurotypical children and adults, confirming convergent validity (Lenoir et al., 2022). Yet, these studies did not evaluate the measure's structural validity and limited information about content development exists, both vital components in psychometric measure development (Prinsen et al., 2018).

Identifying and aligning actions with one's values is a core aspect of ACT, yet limited values measures exist. Reilly et al. (2019) reviewed values measures and identified limited utilisation of the Bulls‐Eye Values Survey (BEVS; Lundgren et al., 2012) in CYP studies, citing studies used small sample sizes and lacked internal consistency evaluation. Concerns about BEVS's sensitivity to detect change were also highlighted. One study in the review adapted the Valued Living Questionnaire (VLQ; Wilson et al., 2010) for an adolescent ACT interventions, which showed acceptable reliability but lacked details on the adaptation process.

### Initial study: Cognitive interviewing & expert consultation

Francis et al. (2016) developed the Comprehensive Assessment of Acceptance and Commitment Therapy (CompACT) measure to address limitations in existing measures and capture multiple psychological flexibility subprocesses. The CompACT reliably and accurately measures three overarching ACT processes: openness to experience (acceptance; cognitive defusion), behavioural awareness (present moment awareness; self as context), and valued action (values; committed action) (Francis et al., 2016). Currently there is no single, comprehensive measure of these psychological flexibility subprocesses validated for CYP.

In an initial study (Lewis, 2020), cognitive interviewing (CI) was employed to assess the suitability of existing CompACT items for CYP. CI is a qualitative method used to evaluate and improve questionnaire item validity and involves participants ‘thinking aloud’ whilst responding to items, to provide insight into how they arrive at their answers (Willis, 2005). Other CI methods involve verbal probing whereby an interviewer asks participants questions to identity any issues with comprehension, for example, relating to wording, order or format (Willis, 2018). The CYP CI study included 36 participants aged 11–18 (*M* = 15.56), comprising 16 individual and five group interviews.

Lewis (2020) found that CYP had challenges in comprehending and understanding the vocabulary in all 23 adult CompACT items and so an adapted set of items suitable for CYP were generated. These adapted items were then reviewed by 11 experts in psychological flexibility and ACT for CYP to refine their wording. The resulting measure, the CompACT‐Y, included 23 adapted items to measure three overarching processes of psychological flexibility and has not yet been validated.

### Present study: Validation of the CompACT‐Y with CYP

#### Aims and hypotheses

This study aimed to validate the CompACT‐Y in a CYP population to explore the internal factor structure of the measure and whether it assesses the intended psychological flexibility constructs. It was predicted that the Compact‐Y would:

(1) Retain a three‐factor structure consistent with the adult CompACT; ‘openness to experience’, ‘valued action’ and ‘behavioural awareness’, indicating structural validity.
(2) Have strong positive correlations with the CAMM and Valuing Questionnaire (VQ) Progress subscale and strong negative correlations with the AFQ‐Y8 and VQ Obstruction subscale, indicating convergent validity.
(3) Have moderate positive correlations with measures of wellbeing: (i) the Short Warwick‐Edinburgh Mental Well‐being Scale, (ii) World Health Organisation‐Five Well‐Being Index and (iii) prosocial behaviours subscale of the Strengths and Difficulties questionnaire (SDQ), indicating concurrent validity.
(4) Have moderate negative correlations with measures of depression, anxiety, stress (Revised Child, Anxiety and Depression Scale and Perceived Stress Scale) and SDQ behavioural difficulties subscales (Emotional, Hyperactivity, Conduct and Peer Difficulties), indicating concurrent validity.

## METHOD

### Participants

Participants were recruited from six schools across the UK between April 2022 and April 2023. Of 383 participants who gave consent, 334 completed the study. Five participants were ineligible due to age, giving a final sample of 329. Participants were aged between 13 and 18 years old (*M* = 16.25, SD = 1.04). Although the sample was non‐clinical, 109 participants had elevated symptoms of depression and anxiety.

Inclusion criteria for participation were secondary students aged 11–18 years and able to communicate fluently in English. If participants required additional support, school staff were asked to provide this through school established supports plans (i.e., a teaching assistant). Participants were required to have a National Curriculum scale reading and writing at level 3 or above due the need to read and understand large amounts of information.

### Measures

The CompACT‐Y measure was completed first and all subsequent measures were administered in a randomised order to minimise order effects. Table 1 summarises the measures administered alongside the CompACT‐Y.

**TABLE 1:** Summary of measures, associated constructs and internal reliability for measures used in validity analysis.

Measure	Number of items	Measured concept and subscales	Example item	Reliability in present sample
Avoidance and fusion questionnaire youth—8 (AFQ‐Y8)	8	Experiential avoidance and fusion	My thoughts and feelings mess up my life	*α* = 0.87
Child and adolescent mindfulness measure (CAMM)	10	Present moment/mindfulness	I think about things that have happened in the past instead of thinking about things that are happening right now.	*α* = 0.87
Perceived stress scale (PSS)	10	Stress	In the last month, how often have you found that you could not cope with all the things that you had to do?	*α* = 0.89
Revised child, anxiety and depression scale (RCADS‐25)	25 (15 items for anxiety, 10 items for Depression)	Anxiety and depression	Anxiety: I worry that I will suddenly get a scared feeling when there is nothing to be afraid of.	*α* = 0.94
			Depression: Nothing is much fun anymore.	
Short warwick‐edinburgh mental well‐being scale (SWEMWBS)	7	Overall well‐being	I've been feeling useful	*α* = 0.85
Strengths and difficulties questionnaire (SDQ)	25 (5 items per subscale)	*Emotion and behaviour*:		*α* = 0.76
		Emotional symptoms	I have many fears, I am easily scared	
		Conduct problems	I fight a lot. I can make other people do what I want	
		Hyperactivity	I am constantly fidgeting or squirming	
		Peer relationship problems	I have one good friend or more	
		Prosocial behaviour	I often volunteer to help others (parents, teachers, children)	
Valuing questionnaire (VQ)	10 (5 items per subscale)	*Valued living*:		
		Progress subscale	I worked toward my goals even if I didn't feel motivated to	*α* = 0.86
		Obstruction subscale	Difficult thoughts, feelings or memories got in the way of what I really wanted to do	*α* = 0.80
World health organisation‐ five well‐being index (WHO‐5)	5	Mental well‐being	My daily life has been filled with things that interest me	*α* = 0.85

#### CompACT‐Y

The CompACT‐Y (Appendix L) is a 23‐item measure of psychological flexibility for young people, adapted from the adult CompACT (Francis et al., 2016) using CI with CYP and expert feedback (Lewis, 2020). Items on the CompACT‐Y are scored from 0 (Strongly Disagree) to 6 (Strongly Agree) with total scores ranging from 0 to 138, where higher scores indicate greater levels of psychological flexibility. The factor structure of the CompACT‐Y when administered to young people has not previously been tested.

#### Avoidance and fusion questionnaire youth‐8 (AFQ‐Y8; Greco et al., 2008)

The AFQ‐Y8 is a measure of experiential avoidance and fusion for adolescents aged 9 years and above. Respondents are asked to rate each statement on how true it is for them, ranging from 0 (Not at all True) to 4 (Very true). Scores are calculated by summing the items and range from 0 to 32, with higher scores suggesting higher psychological inflexibility.

#### Child and adolescent mindfulness measure (CAMM; Greco et al., 2011)

The CAMM is a measure of mindfulness skills for young people aged 10 years and above. Respondents are asked to consider how true a statement is for them and provide an answer on a five‐point scale from 0 (Never True) to 4 (Always True). Items are reverse scored and summed, with a possible range of 0–40. Higher scores represent higher levels of mindfulness.

#### The short warwick‐edinburgh mental well‐being scale (SWEMWBS; Stewart‐Brown et al., 2011)

The SWEMWBS is a measure of mental well‐being. Respondents are asked to answer the statements based on their experiences over the past 2 weeks and rate them on a five‐point scale from ‘None of the Time’ to ‘All of the Time’. Total scores for the measure range from 7 to 35. Higher scores represent better well‐being.

#### The world health organisation‐ five well‐being index (WHO‐5; WHO, 1998)

The WHO‐5 is a brief questionnaire designed to measure current mental well‐being. Respondents are asked to think about their well‐being over the past 2 weeks to answer statements. Responses to each statement range from 0 (At no time) to 5 (All of the time). Raw scores range from 0 to 25 and can be converted to a final score by multiplying the raw score by 4. Final scores range from 0 to 100, or the worst to the best imaginable well‐being.

#### Revised child, anxiety and depression scale (RCADS‐25; Ebesutani et al., 2012)

The RCADS‐25 is a youth measure of depression and anxiety. Respondents rate statements on a four‐point scale; 0 (Never) to 4 (Always). Scoring of the RCADS‐25 provides a anxiety, depression and overall difficulties score by summing items which correspond to each subscale. The anxiety subscale scores range from 0 to 45 and the depression subscales scores from 0 to 30, with higher scores representing increased anxiety or depression symptoms.

#### Perceived stress scale‐10 (PSS‐10; Cohen et al., 1983)

The PSS‐10 measures stress in individuals aged 12 years and older. Responses to items range from 0 (Never) to 4 (Very Often). To score the measure, four items are reverse scored and then all items summed to provide a total score, which ranges from 0 to 40. Higher scores indicate increased levels of stress.

#### Strengths and difficulties questionnaire (SDQ; Goodman et al., 1998)

The SDQ is an 25‐item emotional and behavioural screening tool for young people. The SDQ consists of 5 subscales. A total difficulties score is obtained by summing the first four subscales. Items are answered from ‘Not True’, ‘Somewhat True’ and ‘Certainly True’, and coded as 0, 1 and 2 respectively to obtain a score. Some items are reverse scored and each subscale score ranges from 0 to 10. A total difficulties score is obtained by summing the first four subscales.

#### Valuing questionnaire (VQ; Smout et al., 2014)

The VQ assesses how consistently an individual is living with their values. Each item is answered on a scale of 0 (Not at all true) to 6 (Completely true). The VQ contains two five‐item subscales. The items for each subscale are summed, with higher scores on the Progress subscale representing greater valued living and higher scores on the Obstruction subscale representing a lack of valued living.

### Data analysis

#### Factor analysis

As the CompACT‐Y has not previously been administered to young people, an Exploratory Factor Analysis (EFA) was conducted to determine the underlying factor structure and item variable correlations (Worthington & Whittaker, 2006). A minimum of 230 participants was required for sufficient sample size, based on a ratio of participants to items of 10:1 (Kyriazos, 2018).

Bartlett and Kaiser‐Meyer‐Olkin (KMO) values were calculated to ensure the suitability of using an EFA. Significant Bartlett's test and KMO value ≥ 0.70 were required. Prior to the EFA, items with corrected item‐total correlations below Nunnally and Bernstein's (1994) recommended threshold (*r* < 0.30) were deemed distinct and removed. Inter‐item correlations of items were calculated to evaluate incremental validity, retaining items with average correlations between 0.15 and 0.50, and removed if correlations exceeded 0.80, which suggests an item is redundant (Clark & Watson, 1995). Factors were extracted via Principal Axis Factoring and Oblimin rotation method using three approaches (1) retaining factors with an eigenvalue >1 (Kaiser method; Kaiser, 1974), (2) the scree test method (include factors to the left of the elbow in a scree plot; Cattell & Vogelmann, 1977) and (3) parallel factor analysis (PAF) to retain factors with eigenvalues greater than the eigenvalues generated from random data of corresponding sample size (Horn, 1965). Communalities, the proportion of variance in each item that is shared by the retained factors, were assessed and removed if < 0.20 (Child, 2006).

#### Factor validity and reliability

Non‐loading items were removed and item‐total correlations re‐calculated to assess whether the final items included on the CompACT‐Y were conceptually similar to each other. The internal reliability of the CompACT‐Y was assessed using both reliability coefficient (*α*; Cronbach's alpha) and average inter‐item correlations. Cronbach's alpha values were acceptable if above the threshold of *r* > 0.70 (Nunnally & Bernstein, 1994) and average inter‐item correlations were between 0.15 and 0.50 (Briggs & Cheek, 1986).

#### Validity

To further assess the validity of the CompACT‐Y, correlations with the other measures administered were calculated, with a significance value of *p* < 0.05 used to determine significant correlations.

## RESULTS

### Exploratory factor analysis: CompACT‐Y

Using Mahalanobis distance, eight participants were removed as outliers. An additional case was removed due to incomplete data. This provided a final sample of 320 participants to assess the CompACT‐Y's factor structure. Assumptions for the suitability of EFA were met. The determinant value of 0.00024 was above the recommended threshold of 0.0001 (Field, 2018), indicating the absence of multicollinearity. Bartlett's Test of Sphericity was significant (*χ*2 = 2544.66, df = 231, *p* < 0.001) indicating correlation matrix suitability for factor analysis. KMO (0.87) confirmed adequate sample size and item suitability (>0.50).

Applying Nunnally and Bernstein (1994) recommended threshold (*r* < 0.30), item‐total correlations were analysed to determine whether any items were conceptually distinct. This led to one item (2. ‘*Something that is really important to me is to not have upsetting feelings*’) being removed prior to the EFA being conducted. Inter‐item correlations were also examined to identify items which had significant overlap with one another (*r* > 0.80), indicating the item was redundant and lacked incremental validity. None of the remaining items (*n* = 22) met this criteria.

#### CompACT‐Y factor structure

Factor loadings below 0.45 were excluded, as loadings below this are deemed poor (Tabachnick et al., 2013). Also, factors with fewer than three items were considered unstable, as factors of five items with loading >0.50 are considered more desirable (Costello & Osborne, 2005).

Factors of the CompACT‐Y's remaining 22‐items were initially extracted using Kaiser's criterion (Kaiser, 1974). Principle Axis Factoring was undertaken with an Oblimin rotation and factors with eigenvalues >1 were retained. A five‐factor model was suggested using this method, however factor four had only three items load above the 0.45 cut‐off, and only one item loaded to the fifth factor. Six items did not load to any of the suggested five factors on the pattern matrix, and five items cross‐loaded on the structure matrix. Two items had suboptimal loading across both pattern and structure matrices.

Next, an EFA was run on a four‐factor solution based on a scree test in which factors to the left of the elbow are retained. Based on the pattern matrix, factor four had three items load which ranged between 0.56 and 0.75. Eight items were removed based on non‐loading (*n* = 5) and cross‐loadings (*n* = 3) on either the pattern or structure matrix. The four‐factor model was re‐analysed and this made the fourth factor unstable with only two items loading.

A three‐factor solution was run based on the results of parallel factor analysis (PAF; Horn, 1965); three factors had eigenvalues greater than randomly generated eigenvalues of the same sample size. None of the items cross‐loaded in this model, although one non‐loading item was removed (20. ‘*Thoughts are just thoughts – they don't have to control what I do*’). Analysis was conducted on the remaining 21 items, all of which loaded onto a factor on either the pattern or structure matrices. However, the cumulative variance explained by the three factors fell below the recommended 50% (Streiner, 1994). To surpass this threshold, items with the lowest communalities were systematically eliminated until the total explained variance exceeded 50%, resulting in an additional item with a communality of 0.28, being removed (‘*I'm willing to let myself have whatever thoughts and feelings come up*, *without trying to change or avoid them*’). The analysis was then repeated, leading to the removal of another item that failed to *load* (‘*I can accept how I feel without having to change it*’). Table 2 provides a summary of items removed to form the final three factors.

**TABLE 2:** Items removed from the initial 23 items.

Item	Related theoretical ACT construct based on the adult CompACT
Something that is really important to me is to not have upsetting feelings	Openness to experience; acceptance
I'm willing to let myself have whatever thoughts and feelings come up, without trying to change or avoid them	Openness to experience; acceptance
Thoughts are just thoughts – they don't have to control what I do	Openness to experience; cognitive defusion
I can accept how I feel without having to change it	Openness to experience; acceptance

A final 19‐item, three‐factor model (Table 3) was deemed stable as all items loaded on either the pattern or structure matrices, no items cross‐loaded, and each factor had a minimum of three items with loadings >0.50. The KMO Test (0.87) and Bartlett's test of Sphericity (*χ*2 = 2226.30, df = 171, *p* < 0.001) suggested suitability to run EFA and adequate sample size. The final three‐factor model of 19‐items explained 51.94% of the total variance.

**TABLE 3:** The factor loadings of the 19 items of the CompACT‐Y based on the pattern matrix (*n* = 320).

CompACT‐Y item	Three factor solution		
	Factor 1	Factor 2	Factor 3
I can work out what matters to me in life and go after these things	0.48		
I rush through activities that are important to me, without really paying attention^a^			−0.49
I try to distract myself to block out difficult thoughts and feelings^a^		0.64	
I behave in ways that reflect what is important to me	0.53		
I get so tangled up in my thoughts that I don't do the things that really matter to me^a^		0.38^b^	
I choose to do what's important to me, even if it brings up difficult emotions	0.64		
I tell myself it's wrong to have certain thoughts^a^		0.46	
I find it hard to focus on the thing that I'm doing^a^			−0.54
I live my life in a way that matches what I care about	0.65		
I try to avoid situations that might bring up difficult thoughts or feelings^a^		0.68	
Even when I'm doing things that are important to me, I find myself doing them without paying attention^a^			−0.63
I do things that matter to me, even when it is difficult	0.78		
I try hard to block the feelings I don't want^a^		0.59	
I do things without being aware of what I'm doing^a^			−0.78
I can stick with things that I care about, even when it's difficult	0.71		
I avoid things that are important to me, if there is a risk that I will feel upset^a^		0.41^b^	
I often seem to do things without much awareness of what I'm doing^a^			−0.81
My values are really reflected in my behaviour	0.56		
I can keep going with something when it is important to me	0.73		

^a^ Denotes a reverse scored item.

^b^ Items below 0.45 threshold on pattern matrix, but exceed on structure matrix.

The content of items in the three‐factor model were explored to define the factors. The retained items were consistent with the subscales suggested by the CompACT (Francis et al., 2016):

- Factor 1: Valued action; eight items relating to engagement in behaviours that reflect progress toward one's values (values and committed action)
- Factor 2: Openness to experience; six items relating to avoidance of and entanglement with unwanted thoughts and feelings (acceptance and cognitive defusion)
- Factor 3: Behavioural awareness; five items relating to engagement in mindless or automatic behaviours (contact with the present moment/mindfulness)

These subscales were significantly related to each other (*rs* = 0.35−0.49) although distinct (i.e., *rs* < 0.50) consistent with psychological flexibility theory.

### Reliability of the CompACT‐Y

Item‐total correlations of the CompACT‐Y (19 items, *N* = 320) were all above the recommended threshold (*r* > 0.30), which suggested retained items were conceptually similar. The Cronbach's alpha of the CompACT‐Y was 0.87, suggesting suitable internal reliability. The Cronbach's alpha values for each subscale were also acceptable; 0.85 for ‘valued action’ (VA), 0.76 for ‘openness to experience’ (OE) and 0.81 for ‘behavioural awareness’ (BA). The average inter‐item correlation across all items was 0.26, which was within the appropriate range of 0.15 and 0.50, and no individual item had an average inter‐item correlation below 0.15 (Briggs & Cheek, 1986).

### Validity of the CompACT‐Y

Table 4 summarises correlations between the CompACT‐Y total score (19‐items) and subscales with measures of similar and distinct concepts.

**TABLE 4:** Correlations between the CompACT‐Y total and subscale scores and other measures (*n* = 308).

Measure	Correlation (*r)*			
	CompACT‐Y total score	CompACT‐Y VA subscale	CompACT‐Y OE subscale	CompACT‐Y BA subscale
AFQ‐Y8	−0.61**	−0.41**	−0.56**	−0.47**
CAMM	0.66**	0.39**	0.63**	0.54**
VQ Progress	0.62**	0.65**	0.42**	0.37**
VQ obstruction	−0.64**	−0.41**	−0.58**	−0.51**
RCADS total	−0.65**	−0.44**	−0.55**	−0.54**
RCADS depression	−0.66**	−0.49**	−0.50**	−0.54**
RCADS anxiety	−0.58**	−0.37**	−0.54**	−0.45**
SWEMWBS	0.65**	0.57**	0.49**	0.44**
WHO‐5	0.57**	0.49**	0.47**	0.36**
PSS‐10	−0.60**	−0.41**	−0.55**	−0.46**
SDQ total difficulties	−0.63**	−0.47**	−0.45**	−0.56**
SDQ emotional	−0.58**	−0.43**	−0.56**	−0.37**
SDQ hyperactivity	−0.54**	−0.40**	−0.29**	−0.60**
SDQ conduct	−0.25**	−0.20**	0.04	−0.35**
SDQ Peer difficulties	−0.26**	−0.19**	−0.24**	−0.17**
SDQ Prosocial	0.17**	0.30**	−0.05	0.13*

Abbreviations: BA, Behavioural Awareness; OE, Openness to experience; VA, Valued Action.

*p < 0.05, **p < 0.001.

#### Convergent validity

As expected, the CompACT‐Y had a significant strong negative correlation with the AFQ‐Y8 (*r* = −0.61) and a significant strong correlation with the CAMM (*r* = 0.66). The CompACT‐Y showed significant strong positive/negative correlations with the progress and obstruction subscales of the VQ, respectively (*r* = 0.62 and −0.64).

All three subscales had significant correlations with the other measures of conceptually similar constructs (AFQ‐Y8, CAMM, VQ). The OE subscale had the highest correlations with the AFQ‐Y8 (*r* = −0.56), the CAMM (*r* = 0.63) and VA obstruction subscale (*r* = −0.58). The VA subscale had a strong correlation with the VQ progress subscale as expected (*r* = 0.65).

#### Concurrent validity

The CompACT‐Y total score had significant negative correlations with the RCADS‐25 depression (*r* = −0.66), anxiety anxiety (*r* = ‐0.58) and total (*r* = −0.65) scores. The OE subscale had the highest correlations with the RCADS total and anxiety subscales (*rs* = −0.54 – −0.55). The BA subscale had the highest correlation with the RCADS‐25 depression subscale (*r* −0.54).

Significant positive correlations were found between the CompACT‐Y and the SWEMWBS (*r* = 0.65) and WHO‐5 (*r* = 0.57), and negative correlation with the PSS‐10 (*r* = −0.60). The VA subscale correlated most strongly with the SWEMWBS (*r* = 0.57) and WHO‐5 (*r* = 0.49). All three subscales had moderate significant negative correlations with PPS‐10 (*rs* = −0.41 to −0.55). These suggest the CompACT‐Y has good concurrent validity with measures of distinct concepts in line with psychological flexibility theory.

The CompACT‐Y had a strong negative correlation with the SDQ total score (*r* = −0.63); lower scores on the SDQ indicate fewer difficulties. The CompACT‐Y had moderate negative correlations with the emotional (*r* = −0.58) and hyperactivity (*r* = −0.54) subscales of the SDQ, and weak significant correlations with the conduct (*r* = −0.25), peer difficulties (*r* = −0.26) and prosocial behaviour subscales (*r* = 0.17). The moderate to strong correlations between the SDQ total difficulties, emotional and hyperactivity subscales and the CompACT‐Y indicate that higher psychological flexibility is associated with lower total emotional and hyperactivity difficulties on the SDQ. Overall, these findings suggest acceptable concurrent validity of the CompACT‐Y.

The CompACT‐Y OE subscale had the highest correlation with the SDQ emotional (*r* = −0.56) and peer difficulties (*r* = −0.24), whilst the BA subscale had the highest correlation with the SDQ hyperactivity (*r* = −0.60) and conduct (*r* = −0.35) and the VA subscale with the SDQ prosocial behaviour subscale (*r* = 0.30). These results indicate good concurrent validity for the subscales: when OE scores increase, emotional and peer difficulties decrease, as individuals become more aware of their actions (BA), hyperactivity and conduct difficulties decrease, and as VA increases prosocial behaviours also increase.

## DISCUSSION

The present study aimed to validate the CompACT‐Y, a youth version of an adult psychological flexibility measure (CompACT; Francis et al., 2016). This was achieved by administering the CompACT‐Y alongside other established measures related to psychological flexibility, mental health, well‐being and behaviour. The CompACT‐Y was evaluated to determine its factor structure, validity and reliability.

The EFA process resulted in a 19‐item measure of psychological flexibility, with items based on acceptance, cognitive defusion, mindfulness, values and committed action. In support of hypothesis (1), a three‐factor structure was most stable, consistent with both psychological flexibility theory and the adult CompACT. Similarly, hypothesis (2) was confirmed as the CompACT‐Y total score significantly correlated as predicted with convergent measures of the CAMM, AFQ‐Y8 and VQ. Finally, hypotheses (3) and (4) were also confirmed, as the CompACT‐Y was significantly correlated as predicted with the measures used to assess concurrent validity.

At the subscale level, all three CompACT‐Y subscales (VA, OE and BA) had appropriate internal consistency (*α* > 0.70) which indicates the items in each subscale reliably measure the intended psychological flexibility processes. The CompACT‐Y subscales mostly converged with other ACT processes measures as predicted; the VA subscale had the strongest correlation with the VQ progress subscale, a similar measure of acting in line with one's values, and the OE subscale had the strongest negative correlation with the AFQ‐Y8. This is consistent with psychological flexibility theory, as the OE subscale measures acceptance and cognitive defusion, whilst the AFQ‐Y8 is a measure of experiential avoidance and cognitive fusion.

Unexpectedly, the CompACT‐Y VA subscale had the lowest correlation with the VQ obstruction subscale. In the original validation paper, Smout et al. (2014) found that the VQ obstruction subscale correlated less with another measure of valued living compared to the progress subscale, as was found in the present study. Furthermore, the VQ obstruction subscale exhibited stronger correlations with the AAQ‐II and a mindfulness measure in the validation paper. Similarly, the CompACT‐Y OE and BA subscale showed stronger correlations with the VQ obstruction subscale compared to the CompACT‐Y VA subscale. Other research suggests psychological flexibility and inflexibility are not necessarily opposites (Rogge et al., 2019), and measuring valued‐action may differ from measuring valued‐*inaction*.

In terms of concurrent validity, as expected, all three subscales of the CompACT‐Y were negatively correlated with measures of depression and anxiety (RCADS) and stress (PSS‐10), and positively correlated with measures of well‐being (SWEMWBS, WHO‐5). These findings are consistent with other research (Tyndall et al., 2020) which suggests that higher psychological flexibility leads to lower symptoms of mental health difficulties and better well‐being.

While the CompACT‐Y's final items cover the three overarching ACT processes (openness to experience, behavioural awareness, and valued action), it lacks representation of self‐as‐context, a challenge also faced by the adult CompACT (Francis et al., 2016) due to the complexities of operationalising this concept. Francis et al. (2016) highlighted the reliance on metaphors in ACT to convey self‐as‐context, making it challenging for translation into psychometric measures. The absence of self‐as‐context items in the CompACT‐Y reflects a broader issue of measuring this construct in adolescent ACT research (Godbee & Kangas, 2020; Moran et al., 2018). Additionally, the final CompACT‐Y retained only one of two items related to cognitive defusion from the original 23‐item version which may reflect difficulties YP had in understanding the phrasing of the item (Lewis, 2020).

Although the VQ has not previously been validated in adolescents, alternative validated values measures for CYP are limited (Reilly et al., 2019). Despite this, moderate/strong correlations between the CompACT‐Y VA subscale and VQ subscales was found, as predicted; positively with the VQ progress subscale (measuring action towards values), and negatively with the VQ obstruction subscale (measuring disruptions to living by values). These correlations suggest that the CompACT‐Y VA subscale is a valid measure of values and committed action.

### Implications

The present study provides a possible solution to some of the issues previously highlighted regarding research on ACT for CYP. As the CompACT‐Y has subscales measuring VA, OE and BA which can be examined separately, it offers the ability to explore changes in psychological flexibility subprocesses that might result from ACT interventions. This will enable researchers to examine which ACT components are most predictive of changes in outcomes (Fledderus et al., 2013), which has so far been limited by the availability of a validated measures of psychological flexibility subprocesses. Additionally, the CompACT‐Y is the first comprehensive measure of psychological flexibility processes in adolescents to have both its factor structure and validity examined, as well as having items developed based on a robust content development process (i.e., CI). Future research will be able to utilise the measure in ACT research, addressing issues of multiple measures being required and inconsistency across studies (Fang & Ding, 2020). This will enable data between studies to be pooled and support meta‐analyses to be conducted to establish the evidence‐base of ACT interventions for CYP.

### Strength and limitations

When interpreting the findings of the present study, there are limitations to note. Firstly, the study's sample was restricted to individuals aged 13 and above and lacked diversity, predominantly consisting of White‐British and female participants. Secondly, this study relied on self‐report measures, which are susceptible to response bias and socially desirable responding (Camerini & Schulz, 2018). Thirdly, the inclusion of both positively and negatively worded items in the CompACT‐Y could lead to response confusion, potentially impacting the factor structure and reliability of the instrument (Chyung et al., 2018; Kam, 2023); however, this was weighed against the benefit of including both positively and negatively worded items for limiting acquiescence bias (Mayerl & Giehl, 2018). Despite these limitations, the study identified a theoretically congruent factor structure for the CompACT‐Y and confirmed all hypotheses.

The strengths of this study include the threshold used for factor loadings to be included (0.45) which was stringent enough so items retained are considered more relevant to the final factor structure. Also, the sample size (*N* = 329) was greater than the minimum required (*n* = 230) to provide adequate statistical power for data analyses. Finally, an extensive range of conceptually similar and distinct measures were used to validate the CompACT‐Y. As the main aim of ACT is not solely the reduction of mental health symptoms (Harris, 2006), other constructs such as well‐being, valued living and quality of life are important (Ong et al., 2020). There are also recommendations for ACT research to distinguish process from symptom measures and to compare process measures against behavioural outcomes (Arch et al., 2022). The SDQ, which assesses various behavioural difficulties including peer problems, conduct, and prosocial behaviour, enabled this recommendation to be met.

Content validity ensures that a measure accurately reflects the construct of interest (Mokkink et al., 2010), and that items are relevant to the intended construct and comprehensible to the target population (Prinsen et al., 2018). The CompACT‐Y, unlike measures like AFQ‐Y, CAMM, PsyFlex‐A, and CPFQ, underwent CI and expert consultation to ensure language clarity and construct alignment with the adolescent target group. In contrast, other measures lack sufficient content validity assessment in CYP, risking items not being relevant or meaningful. The CompACT‐Y addressed the essential areas of structural validity and internal consistency, as recommended for outcome measurement development (Prinsen et al., 2018).

## CONCLUSION

To conclude, this study validated the CompACT‐Y, a 19‐item psychological flexibility measure for adolescents, confirming a stable three‐factor structure aligned with psychological flexibility and ACT theory. Convergent validity with similar measures and concurrent validity with mental health indicators support its robustness as an adolescent outcome measure. The CompACT‐Y, addressing research and clinical needs, allows measurement of psychological flexibility subprocesses to ensure ACT interventions target specific aspects as required, reduces patient burden, and enables exploration of associations between psychological flexibility subprocesses and clinical outcomes. Further research, especially in diverse adolescent populations aged 11–13, is needed to confirm its factor structure and validity.

## AUTHOR CONTRIBUTIONS

**Alex Morey**: Conceptualization; data curation; formal analysis; investigation; methodology; project administration; resources; software; validation; visualization; writing – original draft; writing – review & editing. **Victoria Samuel**: Conceptualization; data curation; formal analysis; investigation; methodology; resources; supervision; validation; visualization; writing – original draft; writing – review & editing. **Matthew Lewis**: Investigation; project administration; formal analysis; methodology. **Marc Williams**: Data curation; formal analysis; methodology; supervision; validation; visualization; writing – original draft; writing – review & editing.

## CONFLICT OF INTEREST STATEMENT

The authors declare no conflicts of interest.

## ETHICAL CONSIDERATIONS

Ethical approval was obtained from Cardiff University School of Psychology Research Ethics Committee and informed consent obtained for all participants.

## Data Availability

The data that support the findings of this study are available from the corresponding author upon reasonable request.

## References

[jcv212271-bib-0001] Arch, J. J. , Fishbein, J. N. , Finkelstein, L. B. , & Luoma, J. B. (2022). Acceptance and commitment therapy processes and mediation: Challenges and how to address them. Behavior Therapy, 54(6), 971–988. 10.1016/j.beth.2022.07.005 37863588 PMC10665126

[jcv212271-bib-0002] Bachmann, K. , Hinman, J. M. , Yi, Z. , & Dixon, M. R. (2021). Evaluating the convergent and divergent validity of the Children’s psychological flexibility questionnaire (CPFQ) among children with autism. Advances in Neurodevelopmental Disorders, 5(3), 298–303. 10.1007/s41252-021-00206-w

[jcv212271-bib-0003] Bond, F. W. , Hayes, S. C. , Baer, R. A. , Carpenter, K. M. , Guenole, N. , Orcutt, H. K. , Waltz, T. , & Zettle, R. D. (2011). Preliminary psychometric properties of the acceptance and action questionnaire‐II: A revised measure of psychological inflexibility and experiential avoidance. Behavior Therapy, 42(4), 676–688. 10.1016/j.beth.2011.03.007 22035996

[jcv212271-bib-0004] Briggs, S. R. , & Cheek, J. M. (1986). The role of factor analysis in the development and evaluation of personality scales. Journal of Personality, 54(1), 106–148. 10.1111/j.1467-6494.1986.tb00391.x

[jcv212271-bib-0005] Camerini, A.‐L. , & Schulz, P. J. (2018). Social desirability bias in child‐report social well‐being: Evaluation of the Children’s social desirability Short scale using item response theory and examination of its impact on self‐report family and peer relationships. Child Indicators Research, 11(4), 1159–1174. 10.1007/s12187-017-9472-9

[jcv212271-bib-0006] Cattell, R. B. , & Vogelmann, S. (1977). A comprehensive trial of the scree and kg criteria for determining the number of factors. Multivariate Behavioral Research, 12(3), 289–325. 10.1207/s15327906mbr1203_2 26804294

[jcv212271-bib-0007] Cherry, K. M. , Hoeven, E. V. , Patterson, T. S. , & Lumley, M. N. (2021). Defining and measuring “psychological flexibility”: A narrative scoping review of diverse flexibility and rigidity constructs and perspectives. Clinical Psychology Review, 84, 101973. 10.1016/j.cpr.2021.101973 33550157

[jcv212271-bib-0008] Child, D. (2006). The essentials of factor analysis (3rd ed.). Continuum International Publishing Group Ltd.

[jcv212271-bib-0009] Chyung, S. Y. , Barkin, J. R. , & Shamsy, J. A. (2018). Evidence‐based Survey design: The use of negatively worded items in surveys. Performance Improvement, 57(3), 16–25. 10.1002/pfi.21749

[jcv212271-bib-0010] Clark, L. A. , & Watson, D. (1995). Constructing validity: Basic issues in objective scale development. Psychological Assessment, 7(3), 309–319. 10.1037/1040-3590.7.3.309

[jcv212271-bib-0011] Cohen, S. , Kamarck, T. , & Mermelstein, R. (1983). A global measure of perceived stress. Journal of Health and Social Behavior, 24(4), 385–396. 10.2307/2136404 6668417

[jcv212271-bib-0012] Costello, A. B. , & Osborne, J. (2005). Best practices in exploratory factor analysis: Four recommendations for getting the most from your analysis. 10.7275/JYJ1-4868

[jcv212271-bib-0013] Dawson, D. L. , & Golijani‐Moghaddam, N. (2020). COVID‐19: Psychological flexibility, coping, mental health, and wellbeing in the UK during the pandemic. Journal of Contextual Behavioral Science, 17, 126–134. 10.1016/j.jcbs.2020.07.010 32834970 PMC7392106

[jcv212271-bib-0014] Demkowicz, O. , Ashworth, E. , Mansfield, R. , Stapley, E. , Miles, H. , Hayes, D. , Burrell, K. , Moore, A. , & Deighton, J. (2020). Children and young people’s experiences of completing mental health and wellbeing measures for research: Learning from two school‐based pilot projects. Child and Adolescent Psychiatry and Mental Health, 14(1), 35. 10.1186/s13034-020-00341-7 32973921 PMC7495852

[jcv212271-bib-0015] Doorley, J. D. , Goodman, F. R. , Kelso, K. C. , & Kashdan, T. B. (2020). Psychological flexibility: What we know, what we do not know, and what we think we know. Social and Personality Psychology Compass, 14(12), 125666. 10.1111/spc3.12566

[jcv212271-bib-0016] Ebesutani, C. , Reise, S. P. , Chorpita, B. F. , Ale, C. , Regan, J. , Young, J. , Higa‐McMillan, C. , & Weisz, J. R. (2012). The Revised Child Anxiety and Depression Scale‐Short Version: Scale reduction via exploratory bifactor modeling of the broad anxiety factor. Psychological Assessment, 24(4), 833–845. 10.1037/a0027283 22329531

[jcv212271-bib-0017] Fang, S. , & Ding, D. (2020). A meta‐analysis of the efficacy of acceptance and commitment therapy for children. Journal of Contextual Behavioral Science, 15, 225–234. 10.1016/j.jcbs.2020.01.007

[jcv212271-bib-0018] Field, A. (2018). Discovering statistics using IBM SPSS statistics (5th ed., North American edition). Sage Publications Inc.

[jcv212271-bib-0019] Fledderus, M. , Bohlmeijer, E. T. , Fox, J.‐P. , Schreurs, K. M. G. , & Spinhoven, P. (2013). The role of psychological flexibility in a self‐help acceptance and commitment therapy intervention for psychological distress in a randomized controlled trial. Behaviour Research and Therapy, 51(3), 142–151. 10.1016/j.brat.2012.11.007 23337183

[jcv212271-bib-0020] Fonseca, S. , Trindade, I. A. , Mendes, A. L. , & Ferreira, C. (2020). The buffer role of psychological flexibility against the impact of major life events on depression symptoms. Clinical Psychologist, 24(1), 82–90. 10.1111/cp.12194

[jcv212271-bib-0021] Francis, A. W. , Dawson, D. L. , & Golijani‐Moghaddam, N. (2016). The development and validation of the comprehensive assessment of acceptance and commitment therapy processes (CompACT). Journal of Contextual Behavioral Science, 5(3), 134–145. 10.1016/j.jcbs.2016.05.003

[jcv212271-bib-0022] Gloster, A. T. , Block, V. J. , Klotsche, J. , Villanueva, J. , Rinner, M. T. B. , Benoy, C. , Walter, M. , Karekla, M. , & Bader, K. (2021). Psy‐flex: A contextually sensitive measure of psychological flexibility. Journal of Contextual Behavioral Science, 22, 13–23. 10.1016/j.jcbs.2021.09.001

[jcv212271-bib-0023] Gloster, A. T. , Meyer, A. H. , & Lieb, R. (2017). Psychological flexibility as a malleable public health target: Evidence from a representative sample. Journal of Contextual Behavioral Science, 6(2), 166–171. 10.1016/j.jcbs.2017.02.003

[jcv212271-bib-0024] Godbee, M. , & Kangas, M. (2020). The relationship between flexible perspective taking and emotional well‐being: A systematic review of the “self‐as‐context” component of acceptance and commitment therapy. Behavior Therapy, 51(6), 917–932. 10.1016/j.beth.2019.12.010 33051034

[jcv212271-bib-0025] Goodman, R. , Meltzer, H. , & Bailey, V. (1998). The strengths and difficulties questionnaire: A pilot study on the validity of the self‐report version. European Child & Adolescent Psychiatry, 7(3), 125–130. 10.1007/s007870050057 9826298

[jcv212271-bib-0026] Greco, L. A. , Baer, R. A. , & Smith, G. T. (2011). Assessing mindfulness in children and adolescents: Development and validation of the Child and adolescent mindfulness measure (CAMM). Psychological Assessment, 23(3), 606–614. 10.1037/a0022819 21480722

[jcv212271-bib-0027] Greco, L. A. , Lambert, W. , & Baer, R. A. (2008). Psychological inflexibility in childhood and adolescence: Development and evaluation of the avoidance and fusion questionnaire for youth. Psychological Assessment, 20(2), 93–102. 10.1037/1040-3590.20.2.93 18557686

[jcv212271-bib-0028] Harris, E. , & Samuel, V. (2020). Acceptance and commitment therapy: A systematic literature review of prevention and intervention programs for mental health difficulties in children and young people. Journal of Cognitive Psychotherapy, 34(4), 280–305. 10.1891/JCPSY-D-20-00001 33372124

[jcv212271-bib-0029] Harris, R. (2006). Embracing your demons: An overview of acceptance and commitment therapy. Psychotherapy in Australia, 12(4), 70–76. 10.3316/informit.545561433272993

[jcv212271-bib-0030] Harris, R. (2019). ACT made simple: An easy‐to‐read primer on acceptance and commitment therapy. New Harbinger Publications.

[jcv212271-bib-0031] Hayes, S. C. , Luoma, J. B. , Bond, F. W. , Masuda, A. , & Lillis, J. (2006). Acceptance and commitment therapy: Model, processes and outcomes. Behaviour Research and Therapy, 44(1), 1–25. 10.1016/j.brat.2005.06.006 16300724

[jcv212271-bib-0032] Hayes, S. C. , Strosahl, K. D. , & Wilson, K. G. (1999). Acceptance and commitment therapy: An experiential approach to behavior change. Guilford Press.

[jcv212271-bib-0033] Hayes, S. C. , Strosahl, K. D. , & Wilson, K. G. (2011). Acceptance and commitment therapy. In The process and practice of mindful change (2nd ed.). Guilford Press.

[jcv212271-bib-0034] Horn, J. L. (1965). A rationale and test for the number of factors in factor analysis. Psychometrika, 30(2), 179–185. 10.1007/BF02289447 14306381

[jcv212271-bib-0035] Kaiser, H. F. (1974). An index of factorial simplicity. Psychometrika, 39(1), 31–36. 10.1007/BF02291575

[jcv212271-bib-0036] Kam, C. C. S. (2023). Why do regular and reversed items load on separate factors? Response difficulty vs. Item extremity. Educational and Psychological Measurement, 83(6), 00131644221143972. 10.1177/00131644221143972 PMC10638982 37974659

[jcv212271-bib-0037] Kyriazos, T. A. (2018). Applied psychometrics: Sample size and sample power considerations in factor analysis (EFA, CFA) and SEM in general. Psychology, 09(08), 2207–2230. 10.4236/psych.2018.98126

[jcv212271-bib-0038] Landi, G. , Pakenham, K. I. , Boccolini, G. , Grandi, S. , & Tossani, E. (2020). Health anxiety and mental health outcome during COVID‐19 lockdown in Italy: The mediating and moderating roles of psychological flexibility. Frontiers in Psychology, 11. 10.3389/fpsyg.2020.02195 PMC7488226 32982888

[jcv212271-bib-0039] Lenoir, C. , Hinman, J. M. , Yi, Z. , & Dixon, M. R. (2022). Further examination of the Children’s psychological flexibility questionnaire (CPFQ): Convergent validity and age appropriateness. Advances in Neurodevelopmental Disorders, 6(2), 224–233. 10.1007/s41252-022-00259-5

[jcv212271-bib-0040] Lewis, M. J. (2020). Measuring psychological flexibility in adolescence [dclinpsy, Cardiff university]. https://orca.cardiff.ac.uk/id/eprint/134764/

[jcv212271-bib-0041] Lundgren, T. , Luoma, J. B. , Dahl, J. , Strosahl, K. , & Melin, L. (2012). The bull’s‐eye values Survey: A psychometric evaluation. Cognitive and Behavioral Practice, 19(4), 518–526. 10.1016/j.cbpra.2012.01.004

[jcv212271-bib-0042] Mayerl, J. , & Giehl, C. (2018). A closer look at attitude scales with positive and negative items. Response latency perspectives on measurement quality. Survey Research Methods, 12(3). Article 3. 10.18148/srm/2018.v12i3.7207

[jcv212271-bib-0043] Mokkink, L. B. , Terwee, C. B. , Patrick, D. L. , Alonso, J. , Stratford, P. W. , Knol, D. L. , Bouter, L. M. , & de Vet, H. C. W. (2010). The COSMIN study reached international consensus on taxonomy, terminology, and definitions of measurement properties for health‐related patient‐reported outcomes. Journal of Clinical Epidemiology, 63(7), 737–745. 10.1016/j.jclinepi.2010.02.006 20494804

[jcv212271-bib-0044] Moran, O. , Almada, P. , & McHugh, L. (2018). An investigation into the relationship between the three selves (Self‐as‐Content, Self‐as‐Process and Self‐as‐Context) and mental health in adolescents. Journal of Contextual Behavioral Science, 7, 55–62. 10.1016/j.jcbs.2018.01.002

[jcv212271-bib-0045] Nunnally, J. C. , & Bernstein, I. H. (1994). Psychometric theory (3rd ed.). McGraw‐Hill.

[jcv212271-bib-0046] Ong, C. W. , Pierce, B. G. , Petersen, J. M. , Barney, J. L. , Fruge, J. E. , Levin, M. E. , & Twohig, M. P. (2020). A psychometric comparison of psychological inflexibility measures: Discriminant validity and item performance. Journal of Contextual Behavioral Science, 18, 34–47. 10.1016/j.jcbs.2020.08.007

[jcv212271-bib-0047] Petts, R. A. , Duenas, J. A. , & Gaynor, S. T. (2017). Acceptance and Commitment Therapy for adolescent depression: Application with a diverse and predominantly socioeconomically disadvantaged sample. Journal of Contextual Behavioral Science, 6(2), 134–144. 10.1016/j.jcbs.2017.02.006

[jcv212271-bib-0048] Prinsen, C. A. C. , Mokkink, L. B. , Bouter, L. M. , Alonso, J. , Patrick, D. L. , de Vet, H. C. W. , & Terwee, C. B. (2018). COSMIN guideline for systematic reviews of patient‐reported outcome measures. Quality of Life Research, 27(5), 1147–1157. 10.1007/s11136-018-1798-3 29435801 PMC5891568

[jcv212271-bib-0049] Reilly, E. D. , Ritzert, T. R. , Scoglio, A. A. J. , Mote, J. , Fukuda, S. D. , Ahern, M. E. , & Kelly, M. M. (2019). A systematic review of values measures in acceptance and commitment therapy research. Journal of Contextual Behavioral Science, 12, 290–304. 10.1016/j.jcbs.2018.10.004

[jcv212271-bib-0050] Rochefort, C. , Baldwin, A. S. , & Chmielewski, M. (2018). Experiential avoidance: An examination of the construct validity of the AAQ‐II and MEAQ. Behavior Therapy, 49(3), 435–449. 10.1016/j.beth.2017.08.008 29704971

[jcv212271-bib-0051] Rogge, R. D. , Daks, J. S. , Dubler, B. A. , & Saint, K. J. (2019). It’s all about the process: Examining the convergent validity, conceptual coverage, unique predictive validity, and clinical utility of ACT process measures. Journal of Contextual Behavioral Science, 14, 90–102. 10.1016/j.jcbs.2019.10.001

[jcv212271-bib-0052] Smout, M. , Davies, M. , Burns, N. , & Christie, A. (2014). Development of the valuing questionnaire (VQ). Journal of Contextual Behavioral Science, 3(3), 164–172. 10.1016/j.jcbs.2014.06.001

[jcv212271-bib-0053] Soares, R. , Cunha, M. , Massano‐Cardoso, I. , & Galhardo, A. (2023). Assessing psychological flexibility in adolescents: Validation of PsyFlex‐A. Revista Portuguesa de Investigação Comportamental e Social, 9(1). 10.31211/rpics.2023.9.1.284

[jcv212271-bib-0054] Stewart‐Brown, S. L. , Platt, S. , Tennant, A. , Maheswaran, H. , Parkinson, J. , Weich, S. , Tennant, R. , Taggart, F. , & Clarke, A. (2011). The warwick‐edinburgh mental well‐being scale (WEMWBS): A valid and reliable tool for measuring mental well‐being in diverse populations and projects. Journal of Epidemiology & Community Health, 65(Suppl 2), A38–A39. 10.1136/jech.2011.143586.86

[jcv212271-bib-0055] Streiner, D. L. (1994). Figuring out factors: The use and misuse of factor analysis. Canadian Journal of Psychiatry / La Revue Canadienne de Psychiatrie, 39(3), 135–140. 10.1177/070674379403900303 8033017

[jcv212271-bib-0056] Swain, J. , Hancock, K. , Hainsworth, C. , & Bowman, J. (2015). Mechanisms of change: Exploratory outcomes from a randomised controlled trial of acceptance and commitment therapy for anxious adolescents. Journal of Contextual Behavioral Science, 4(1), 56–67. 10.1016/j.jcbs.2014.09.001

[jcv212271-bib-0057] Tabachnick, B. G. , Fidell, L. S. , & Ullman, J. B. (2013). Using multivariate statistics (7th ed.). Pearson.

[jcv212271-bib-0058] Timko, C. A. , Zucker, N. L. , Herbert, J. D. , Rodriguez, D. , & Merwin, R. M. (2015). An open trial of Acceptance‐based Separated Family Treatment (ASFT) for adolescents with anorexia nervosa. Behaviour Research and Therapy, 69, 63–74. 10.1016/j.brat.2015.03.011 25898341 PMC4869854

[jcv212271-bib-0059] Tyndall, I. , Waldeck, D. , Pancani, L. , Whelan, R. , Roche, B. , & Pereira, A. (2020). Profiles of psychological flexibility: A latent class analysis of the acceptance and commitment therapy model. Behavior Modification, 44(3), 365–393. 10.1177/0145445518820036 30580551

[jcv212271-bib-0060] Willis, G. (2018). Cognitive interviewing in Survey design: State of the science and future directions. In D. L. Vannette & J. A. Krosnick (Eds.), The palgrave handbook of Survey research (pp. 103–107). Springer International Publishing. 10.1007/978-3-319-54395-6_14

[jcv212271-bib-0061] Willis, G. B. (2005). Cognitive interviewing: A tool for improving questionnaire design. Sage Publications.

[jcv212271-bib-0062] Wilson, K. G. , Sandoz, E. K. , Kitchens, J. , & Roberts, M. (2010). The valued living questionnaire: Defining and measuring valued action within a behavioral framework. Psychological Record, 60(2), 249–272. 10.1007/BF03395706

[jcv212271-bib-0063] Wolgast, M. (2014). What does the acceptance and action questionnaire (AAQ‐II) really measure? Behavior Therapy, 45(6), 831–839. 10.1016/j.beth.2014.07.002 25311291

[jcv212271-bib-0064] Wolpert, M. , Cheng, H. , & Deighton, J. (2015). Measurement issues: Review of four patient reported outcome measures: SDQ, RCADS, C/ORS and GBO – their strengths and limitations for clinical use and service evaluation. Child and Adolescent Mental Health, 20(1), 63–70. 10.1111/camh.12065 32680325

[jcv212271-bib-0065] World Health Organization (WHO) . (1998). Wellbeing measures in primary health care/the DEPCARE project: Report on a WHO meeting.

[jcv212271-bib-0066] Worthington, R. L. , & Whittaker, T. A. (2006). Scale development research: A content analysis and recommendations for best practices. The Counseling Psychologist, 34(6), 806–838. 10.1177/0011000006288127

